# The Complex Relationship between Liver Cancer and the Cell Cycle: A Story of Multiple Regulations

**DOI:** 10.3390/cancers6010079

**Published:** 2014-01-13

**Authors:** Xavier Bisteau, Matias J. Caldez, Philipp Kaldis

**Affiliations:** 1 Institute of Molecular and Cell Biology (IMCB), A*STAR (Agency for Science, Technology and Research), 61 Biopolis Drive, Proteos#3-09, Singapore 138673, Singapore; E-Mails: xbisteau@imcb.a-star.edu.sg (X.B.); mjcaldez@imcb.a-star.edu.sg (M.J.C.); 2 National University of Singapore (NUS), Department of Biochemistry, Singapore 117597, Singapore

**Keywords:** cell cycle, cancer, liver, hepatocellular carcinoma, cyclin-dependent kinases, CKI, cyclins

## Abstract

The liver acts as a hub for metabolic reactions to keep a homeostatic balance during development and growth. The process of liver cancer development, although poorly understood, is related to different etiologic factors like toxins, alcohol, or viral infection. At the molecular level, liver cancer is characterized by a disruption of cell cycle regulation through many molecular mechanisms. In this review, we focus on the mechanisms underlying the lack of regulation of the cell cycle during liver cancer, focusing mainly on hepatocellular carcinoma (HCC). We also provide a brief summary of novel therapies connected to cell cycle regulation.

## 1. Liver and Cancer

The liver is located at a strategic position in the human body and regulates metabolic homeostasis by producing energy and molecules used by other cells in nearby or very distant tissues. The liver has the unique capacity to regenerate upon injury [1]. If the inflicting agent persists or becomes chronic, the self-repairing ability works as a double-edged sword potentially resulting in cirrhosis, (acute) liver failure, and/or development of liver neoplasia [2,3,4]. Liver cancer is ranked in the top 10 human cancers worldwide and among the top five of cancers in terms of mortality [5,6,7]. More than 70% of primary liver cancer [2,8,9] is presented as hepatocellular carcinoma (HCC) but also comprises primary neoplasms like hepatoblastoma, cholangiocarcinoma, epithelioid hemangioendothelioma, and hemangiosarcoma [2,9]. Secondary liver cancer comprises metastases from distant tumors, commonly from the gastrointestinal tract.

Primary liver cancers display an important epidemiologic trend where the prevalence is related to different risks and etiologic factors in different geographic regions of the world [10]. Many studies suggested that the risk factors related to liver cancer are alcohol abuse, smoking, exposure to aflatoxins, sex, ethnicity, as well as infection by hepatitis B (HBV) and C viruses (HCV) [11,12,13]. Whereas heavy alcohol intake is the main cause of cirrhosis, long-term exposure to aflatoxins and HBV and/or HCV infection increase the frequency of liver cancer development and HCC [14,15,16,17]. For further reading on the epidemiology of liver cancer see [13,18,19].

Whereas different types of primary liver cancer display dissimilar phenotypes and diverse molecular mechanisms, their development processes implicate a combination of several hallmarks of cancer [20]. For example, cell cycle misregulation plays a central role in promoting hepatocarcinogenesis through evasion of growth suppressors, sustaining proliferative signaling, resistance to cell death, potentially acting on chromosome instability, as well as invasiveness and metastasis.

To better understand the relationship between HCC and cell cycle regulation, we will introduce in the following sections the normal and oncogenic cell signaling pathways associated with hepatocyte cell cycle progression, with a focus on (potential) mechanisms of HCC development. Furthermore, with the goal to give insight into potent cell cycle based therapies against HCC, we will dissect cell cycle regulation and its particularities observed in the normal and pathologic liver.

### 1.1. Liver Anatomy and Regeneration

The anatomy of liver is unique as it is divided into lobes that are each connected and supplied by branches of the portal vein, the hepatic artery, and the bile duct [1]. The main metabolic activity of the liver is provided by hepatocytes accounting for 60%–80% of the liver mass while other cells such as biliary epithelial cells, hepatic stellate cells, Kupffer, sinusoidal endothelial cells, and liver-specific NK cells participate in liver maintenance and repair, immune system, or contribute to liver architecture [1,21]. Due to its functions and localization, the liver is exposed to insults from external factors that can induce hepatocyte cell death and liver mass loss, triggering liver regeneration. During this process, which depends on reactivation of transcriptional program leading to exit from quiescence and to enter into the cell cycle of (mostly) hepatocytes, the liver maintains all its metabolic functions [22]. The relative independence of each lobe makes it possible to dissect each liver lobe following a protocol called Partial Hepatectomy (PHx) in which generally 70% of the rodent liver mass is removed surgically. PHx can be used as a model of *in vivo* cell growth since the liver will recover its original mass after a short time by *compensatory hyperplasia* [23,24].

### 1.2. Molecular Mechanisms of Hepatocellular Carcinoma

Because of the prevalence, HCC is the most studied primary liver cancer. Several different histological subtypes are known such as scirrhous HCC, fibrolamellar carcinoma, combined HCC-cholangiocarcinoma (HCC-CC), sarcomatoid HCC, undifferentiated carcinoma, lymphoepithelioma-like HCC, clear cell HCC, diffuse cirrhosis-like HCC, steatohepatitic HCC, transitional liver cell tumor, and CAP carcinoma [25]. The lack of useful molecular markers to classify HCC aggressiveness hereby complicates clinical analyses to stage patient’s outcomes [26]. To generate a classification protocol integrating histological information and patient outcomes that can be applied to develop clinical trials, the American (AASLD), European (EASL), and Asian Pacific associations for the study of the liver defined guidelines establishing standards in the selection of prognostic factors related to the liver function, tumor progression, and the general health status [27,28,29]. Accordingly, several classification protocols are available for HCC [29] whereby the Barcelona Clinic Liver Cancer (BCLC) classification has emerged as the standard classification validated by expert panels in local European and American populations [30,31]. In order to uncover reliable molecular markers related to HCC, many groups have applied high-throughput technologies to HCC but obtained results that were unable to provide enough resolution to stage HCC correctly [32,33,34]. Nevertheless, these efforts have helped to understand tumor development at the molecular level but there is still a need to integrate the high-throughput data for a more comprehensive understanding of the disease.

**Figure 1:**
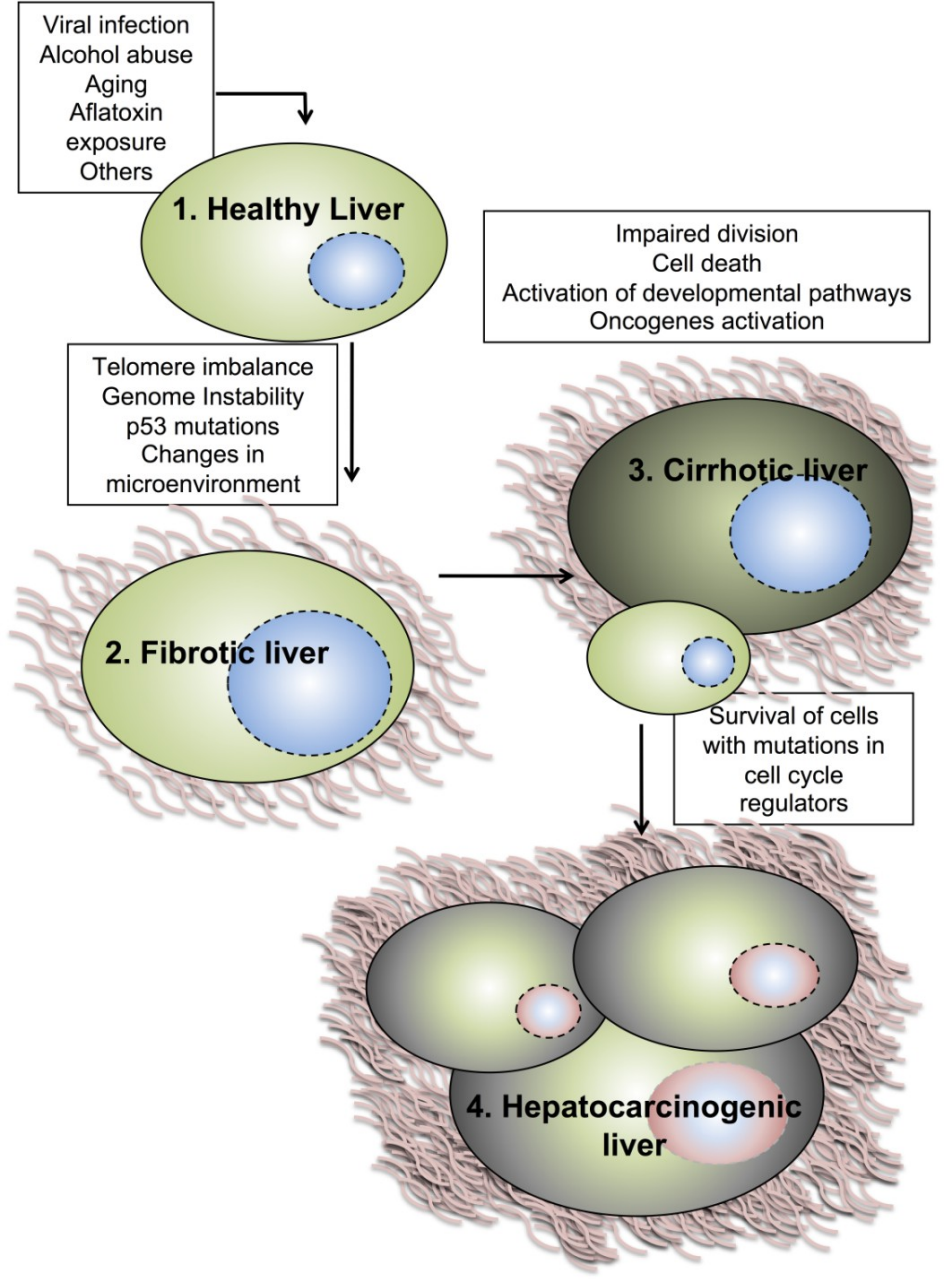
Model for the development of primary liver tumors. Several risk and etiologic factors can lead to development of primary tumors in the liver. HCV and HBV infection, heavy alcohol intake, exposure to aflatoxin, drugs, and others can lead to cycles of damage-cell death-regeneration in the liver. This, together with genome instability and changes in the microenvironment, can create a scenario where hepatocytes start to activate oncogenic and developmental pathways. Only those hepatocytes with mutations in cell cycle regulators will survive and divide, resulting in malignancy and metastases.

Development of liver tumors and their evolution to HCC is a multi-step process where different HCC-etiologies provoke continuous rounds of hepatocytes damage and regeneration (Figure 1). These cycles of damage-death-regeneration lead to collagen accumulation contributing to liver fibrosis. Over an extended time, this triggers a cirrhotic state considered as a pathological state of the liver [35] whose lesions can progress to a pre-malignant state producing dysplastic nodules. Later, these nodules will evolve to HCC invading the surrounding stroma and occasionally generating metastatic events [36]. In this context, the involved molecular mechanisms include different cellular alterations as well as modifications of microenvironment of the liver. Among the first cellular-intrinsic alterations occurring during hepatotumorigenesis, telomere shortening gives rise to a loss of cell cycle checkpoint regulation impairing hepatocytes proliferation. At the transition of premalignant lesions to HCC, this effect is rapidly reversed by telomerase activation and up-regulation of telomerase reverse transcriptase (TERT) in 90% of human HCC [18]. On the other hand, the underlying fibrotic state of the liver can create a microenvironment where cytokines secreted by myofibroblasts and infiltrating immune cells select for hepatocytes carrying mutations to survive, to clonally proliferate, and subsequently to develop into tumors [19]. Transcriptional analyses of liver tumors revealed alterations of several molecular pathways during cancer development implicated in cell proliferation, cell cycle regulation, apoptosis, angiogenesis, cell signaling, metabolism, and immune response (particularly in HCC with HBV/HCV infection) [19,20,33,37,38,39]. Persistent intracellular signaling induced by oncogene or tumor suppressor dysfunction seems to be the main mechanism for tumor development stimulating cell cycle progression and enhancing cell survival like in other tumor types too.

In 20%–80% of HCC, signaling cascades such as the Wnt, Met, or Akt are frequently found altered alone or in combination. These can be associated or not with alterations in the Myc, p53, or pRb pathway [40,41]. Activation of the canonical Wnt pathway is the most frequent alterations observed [37]. In the normal liver, Wnt signaling is tightly regulated. In the absence of Wnt ligand, β-catenin is phosphorylated and degraded by a complex (GSK3, Ck1-α, APC, and Axin). Binding of Wnt ligand to Frizzled receptors (FZD) recruits, after a phosphorylation cascade, Axin1 and GSK3β to the plasma membrane resulting in β-catenin accumulation in the cytoplasm. Its subsequent translocation to the nucleus initiates transcription of target genes such as cyclin D1 via its interaction with TCF/LEF (T-cell factor/Lymphoid enhancer factor) transcription factors [42,43,44]. Mutations in the β-catenin gene (CTNNB1) is the second most frequent mutation observed after the p53 tumor suppressor (TP53) [45]. Although often mutually exclusive, inactivating APC or AXIN mutations and overexpression of FZD receptors with Wnt ligands occur in 33%–67% of HCCs [39,44,46].

Originally associated with poor prognosis of HCC, genetic analyses have revealed that c-Myc is commonly overexpressed in addition to genomic amplification in up to 70% of viral and alcohol-related HCC [47,48,49]. Sustained activation of c-Myc is sufficient to induce hepatocarcinogenesis while its inactivation induces massive regression of c-Myc-induced liver cancers resuming a physiological program and differentiation of tumors cells into normal hepatocytes in genetic mouse models [50]. An extensive number of genes can be regulated by c-Myc affecting ribosomal and mitochondrial biogenesis, glucose and glutamine metabolism, lipid synthesis, cytoskeleton, and cell cycle progression [51].

Like other pathways, the c-Met protooncogene is frequently deregulated in the context of HCC. Stimulated by the hepatocyte growth factor (HGF), the c-Met receptor with tyrosine kinase activity activates a broad range of cascades (PI3K, Stat, Ras/Erk, *etc.*) inducing numerous responses such as cell survival and proliferation or cell motility and invasion [39,52,53]. The mechanisms of deregulation of the c‑Met receptor are wide-ranging and include overexpression and mutation of the receptor leading to constitutive kinase activation in the presence or absence of gene amplification as well as paracrine or autocrine activation of c-Met by HGF [53].

Finally, direct cell cycle deregulation, especially in the pRb pathway appears in more than two thirds of HCC by various mechanisms including gene amplification, deletion, silencing or mutations leading to up or down regulation of associated cell cycle transcripts and/or proteins. Furthermore, since cell cycle regulators connect numerous signaling pathways and integrate their oncogenic perturbations, all HCC display deregulations of cell cycle checkpoints. Oncogenic alterations of the cell cycle are well known to accelerate cell proliferation and are required for tumorigenesis but reveal also unexpected effects and particularities in HCC as described below.

## 2. Hepatocyte Cell Cycle Progression

Under normal conditions, hepatocytes are fully differentiated and do not proliferate but are able, upon injury, to exit their quiescent state (G0), enter the cell cycle, and progress through the four different cell cycle phases (G1, S, G2, M) to finally divide. The progression of hepatocytes through the cell cycle follows a well-characterized cascade of events found in all proliferating cells.

Progression through the different cell cycle phases is controlled by the activity of cyclin-dependent kinases (Cdk). Cdks constitute a family of 20 members [54,55] of which five (Cdk1, 2, 3, 4, 6) have been associated with cell cycle control so far. Cdks associate with a cyclin to form an active complex. Cyclins are classified in fifteen types named from A to Y. Some types of cyclins comprise different orthologs such as cyclins A1/2, B1/2/3, D1/2/3, or E1/2. Cdk activity is tightly regulated during the different phases by diverse mechanisms. While the expression of Cdk2/4/6, in contrast to Cdk1, does not substantially vary between phases, time specific expression of cyclins during cell cycle orchestrates Cdk/cyclin complex formation, activation, and resulting activity. In addition, binding of Cdk inhibitor proteins limits Cdk activity. These inhibitors fall into two different families (Cip/Kip and Ink4) based on their inhibitory mechanism. The three members of the Cip/Kip family (p21^Cip1/Waf1/Cdkn1a^, p27^Kip1/Cdkn1b^, p57^Kip2/Cdkn1c^) bind all Cdk/cyclin complexes and inhibit their activity by fitting into the ATP binding site of Cdks but when highly expressed, impair also the activating phosphorylation mediated by the CAK complex (Cdk7/cyclin H/Mat1). Ink4 inhibitors (p16^Cdkn2a/Ink4a^, p15^Cdkn2b/Ink4b^, p18^Cdkn2c/Ink4c^, p19^Cdkn2d/Ink4d^) bind only to Cdk4/6 and modify the conformation impairing cyclin D binding. Therefore, Ink4 inhibitors force cyclin D expression to exceed a threshold in order to associate and activate Cdk4/6 [56].

**Figure 2:**
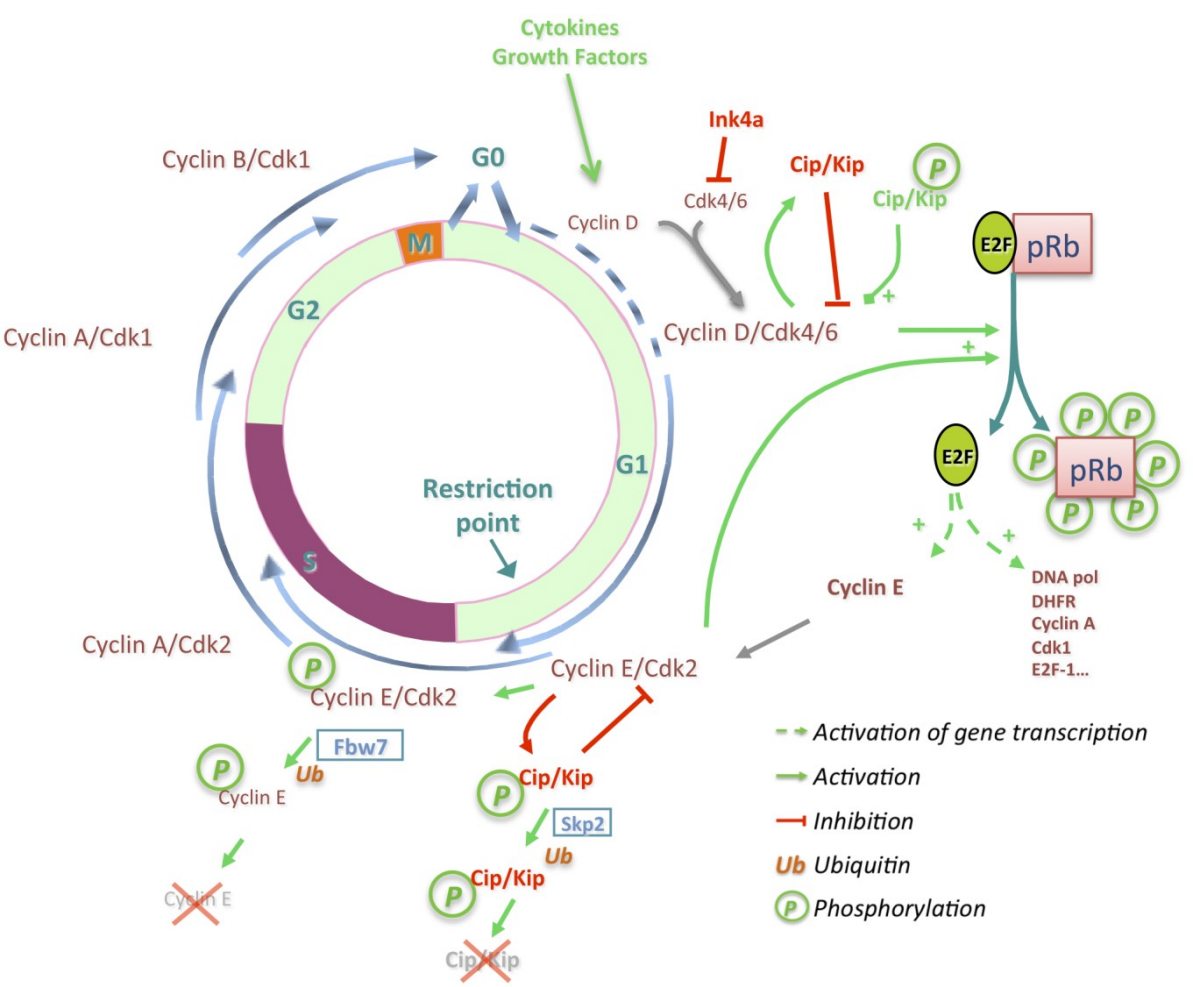
Progression through the different cell cycle phases is under the control of Cdk/cyclin complexes. Ink4 inhibitors impose a threshold on cyclin D expression to bind and activate Cdk4 in G1 phase. Newly formed Cdk4/cyclin D complexes are stabilized by Cip/Kip proteins and phosphorylate p21, changing its inhibitory to an activating state. Cdk4/cyclin D complexes initiate pRb phosphorylation, inducing the release from E2F1 transcription factors and promoting expression of genes implicated in the DNA synthesis phase and S phase progression. Cyclin E associates with Cdk2 enhancing pRb phosphorylation and phosphorylates Cip/Kip proteins leading to their ubiquitination and degradation. Successive association of Cdk2 with cyclin A, after the ubiquitination and degradation of cyclin E by Fbw7, leads to S phase completion and entry into G2 phase. Association of Cdk1 with cyclin A followed by cyclin B1 permits G2/M transition, entry in mitosis, and finally cytokinesis.

Elevated expression of Cdk inhibitors results in cell cycle arrest. In the absence of extracellular proliferative stimuli, p27 maintains the quiescent state of hepatocytes, restraining the activity of Cdk complexes, especially of Cdk2 [57,58,59]. In this state, hepatocytes express membrane receptors, which can be activated by a large panel of cytokines (IL-6, TNFα, TGFβ…) or growth factors (EGF, HGF…) resulting in the expression of early response genes (see [60] for a detailed review). This allows cell cycle re-entry through the expression of cyclin D1, among a variety of other *de novo* proteins. In contrast to its homolog cyclin D3, which is already expressed during the quiescent state [61], cyclin D1 is strongly induced by cytokines and growth factors in late G1 and is sufficient to promote cell cycle progression in hepatocytes [62,63,64,65] (Figure 2). The consecutive formation of Cdk4/cyclin D1 complexes is quickly followed by activating phosphorylation on Thr172-Cdk4 [66,67,68]. The G1/S transition is promoted after Cdk4/cyclin D dependent-phosphorylation of the retinoblastoma protein (pRb). Together with its two homologs p107 and p130, pRb constitutes the Rb family, called also “pocket” proteins, which repress E2F transcription factors [69,70]. Active Cdk4/cyclin D complexes initiate pRb phosphorylation while newly formed Cdk2/cyclin E enhances pRb inactivation resulting in a conformational change and the release of the associated E2F transcription factors [71,72,73]. This transcriptionally activates the expression of numerous genes implicated in DNA replication or genes involved in cell cycle progression such as cyclin E, A, B, Cdk1, and E2F1. The positive feedback loop linking pRb inactivation, cyclin E expression, and E2F1 release creates a bistable switch (Figure 2), which allows the non-reversible passage through the restriction point (R), the G1/S transition, and cell cycle progression [74].

Although temporal expression of cyclins and Cdk phosphorylation suffice for cell cycle progression, Cdk inhibitors impose a tight regulation on the activity of Cdk/cyclin complexes. p27 is highly expressed during quiescence of hepatocytes but declines after cell cycle entry. In contrast, its homolog p21 is rapidly induced in early G1 phase in hepatocytes [75] contrasting with its known inhibitory effect observed in numerous studies. p21 levels increase like cyclin D1 expression in early G1 to allow the nuclear localization of Cdk4/cyclin D complexes and stabilize their formation without inhibiting their activity [68,76,77,78]. At the G1/S transition, the decrease of free p21 levels is initiated via its sequestration by Cdk4-6/cyclin D complexes priming Cdk2/cyclin E activation [79]. Consequently, p21 is phosphorylated on Ser130 inducing the change from an inhibitor to an activator, promoting the activity of Cdk4/cyclin D complexes. Cdk2/cyclin E complexes enhance the phosphorylation of p21 on Ser130 inducing its ubiquitination and degradation (Figure 2) [68,80]. 

Various observations obtained using mouse models lacking p21 or p27 inhibitors have confirmed their inhibitory effects indicating an acceleration of G1 progression and S phase entry of hepatocytes [57,81]. Once Cdk inhibitors are degraded by the proteasome following their ubiquitination by Skp2 [82,83], cyclin E and cyclin A associate successively with Cdk2 to initiate, maintain, and complete DNA replication. In parallel to the increase of Cdk2 activity, Cdk2/cyclin E auto-phosphorylates cyclin E. Cyclin E is thereafter ubiquitinated by SCF^Fbw7^ resulting in its degradation (Figure 2) [84,85]. In turn, free Cdk2 associates with cyclin A which later will assemble with Cdk1 when Cdk2/cyclin A association reaches a plateau in late S and G2 phase [86]. In addition to its cyclin binding, Wee1 and Myt1 phosphorylate Cdk1, as well as Cdk2, on Thr14 and Tyr15, two inhibitory sites, which are dephosphorylated by Cdc25 and required for Cdk activity. Mitosis is driven by the activity of Cdk1/cyclin B complexes, which phosphorylate numerous substrates to regulate chromosome condensation and segregation and to orchestrate the proper division of cells.

## 3. Deregulations of Cell Cycle Genes in HCC

### 3.1. p16 and pRb: Tumor Suppressors

**Figure 3:**
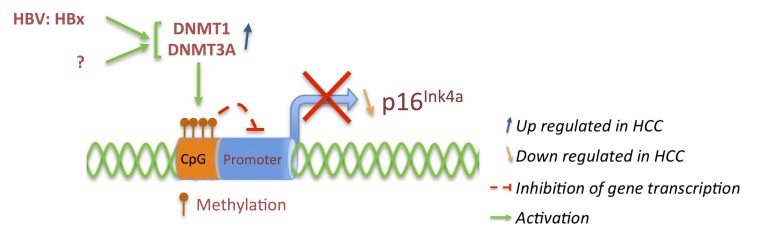
p16^Ink4a^ expression is down regulated in HCC through hypermethylation of CpG islands as a result of increased expression of DNA methyltransferase DNMT1 and DNMT3A associated with hepatitis B virus expression or due to other mechanisms.

The retinoblastoma protein (pRb) is often anomalously expressed in many types of HCC as reported in several studies. Analysis of chromosome region (CR) 13q coding among others for the RB1 gene revealed frequent loss of heterozygosity (LOH), associated with absence of pRb expression in 50% of tested tumors [87] although less was observed in other studies [88,89]. Nevertheless, the expression of pRb varies drastically between tumors, not necessarily correlating with its tumor suppressor activity, especially after truncation or mutation of its gene [87,90]. In contrast, hypermethylation of 5' CpG islands around the p16^Ink4a^ and p14^Arf^ shared promoter is associated with absence of their expression in HCC (Figure 3) [91,92,93]. Homozygous deletions or mutations rendering p16 unable to interact with Cdk4 also exist but remain rare [91,94,95].

Recent observations suggested up regulation of DNA methyltransferases (DNMT1 and DNMT3A) and interaction with hepatitis B virus x protein (HBx) correlating with hypermethylation of p16^Ink4a^ gene (Figure 3) [96]. Expression analysis of DNMT1 and DNMT3A in various HCC cell lines and HCC tumor samples revealed an increase of mRNA and protein levels in HBx positive samples particularly in non-cancerous cases [97]. It is therefore not surprising to detect p16 promoter hypermethylation already at early stages of liver dysplasia as well as in chronic hepatitis and cirrhotic nodules associated with HBV or HCV infection [98,99,100,101]. These results suggest that hepatitis B virus deregulates p16 at early stage inducing gradually hepatocarcinogenesis, while at late stages of HCC p16 gene hypermethylation is selected when HBx expression appears to decrease [97].

**Figure 4:**
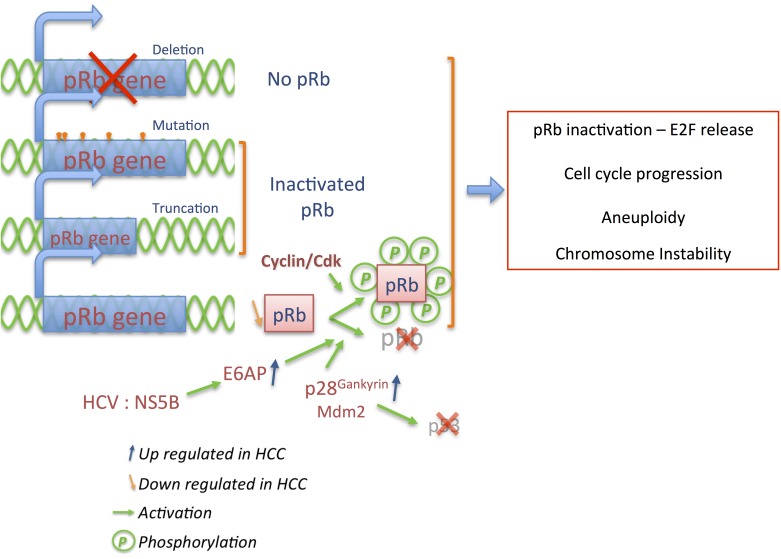
Inactivation of pRb in HCC is complex. Deletion, mutation, or truncation of the pRb gene leads to an inactive form or complete absence of the pRb protein. Overexpression of the E6AP ubiquitin ligase or p28^Gankyrin^ in HCC enhances degradation of pRb while hyperactivity of Cdk/cyclin complexes inactivates pRb by sustained hyperphosphorylation. Those mechanisms impair pRb tumor suppressor activity and induce persistent E2F activity, triggering cell cycle progression as well as aneuploidy and chromosome instability.

Although pRb can be directly inactivated via multiple ways and more often in advanced stage HCC, HCV alters the pRb cascade through a mechanism leading to pRb degradation (Figure 4). The nonstructural protein (NS) of hepatitis C virus, NS5B, affects the proper control of proliferation by interacting with the ubiquitin ligase E6AP which degrades pRb [102,103]. Among other mechanisms independent of HCV, overexpression of p28^gankyrin^ (component of the 19S regulatory cap of the proteasome), was observed in a high proportion of tested HCC (80%–100%), and induces pRb degradation by binding to the pRb LxCxE motif [104,105]. Furthermore, p28^gankyrin^ binds multiple proteins and plays roles in diverse pathways such as NFκB, AKT/PI3K/HIF1α, MDM2, ß-catenin or c-Myc affecting cell proliferation, apoptosis and hypoxia response, or HCC invasiveness and metastasis [106,107,108,109]. Its interaction with the MDM2 ubiquitin ligase facilitates p53 ubiquitination as well as pRb degradation [110,111], strengthening the crosstalk between pRb and p53 stability (Figure 4) [112]. Despite this crosstalk, p53 re-expression induces growth arrest in Hep3B cells lacking pRb [113] indicating that p53 and pRb have complementary effects in hepatocarcinogenesis. Loss of p16^Ink4a^ and pRb expression is inversely correlated, especially during early stage of HCC, although they have also been observed conjointly expressed in poorly differentiated HCC associated with metastasis [114,115].

The loss of proper control of the pRb pathway corresponds to evasion of growth suppressors but can also be associated with other hallmarks of cancer [20]. Hepatic deletion of the pRb gene in mice (Rb^flox/flox^ Alb-Cre) resulted, as expected, in ectopic entry into the cell cycle [116]. However, expression of p107 and p130 pocket proteins rapidly compensates for pRb loss inducing hepatocytes proliferation arrest as confirmed in pocket protein triple knockout mice. In this context, p107 and p130 repress E2F1 targets genes, inhibit proliferation, and liver hyperplasia in adult mice [116] while in their combined absence (pRb, p107, p130 TKO) mice develop liver tumors, with a gene expression signature similar to human HCC [117].

However, loss of pRb has been associated with aberrant ploidy in liver, which combined with carcinogen treatment (diethylnitrosamine—DEN), upregulates expression of genes well associated to chromosome instability [116,118]. In addition, low expression of pRb coupled with genotoxic hepatocarcinogen aflatoxin B1 (AFB1) exposure, does not abrogate the aberrant proliferative response mediated by AFB1, increases DNA double-stranded breaks, mitotic failure, and finally the susceptibility for HCC development [119]. When pRb deletion is combined with hepatic deletion of p53 in addition to DEN treatment, genome instability as well as deregulation of the cell cycle and checkpoint response is further exacerbated. p53^−/−^pRb^−/−^ DKO mice do not develop liver tumors spontaneously due to potent quiescence mechanisms [120] and possibly the presence of p27 [121].

Taken together, these observations contradict the idea that inactivation of Rb alone is sufficient to promote hepatic tumor development as in other tissues. This may indicate that loss of pRb or its inactivation by hyperactivated Cdk/cyclin complexes affects HCC through different mechanisms and rather functions as a proliferative accelerator in the advanced stage tumors.

### 3.2. Cyclins: The Oncogenes

**Figure 5:**
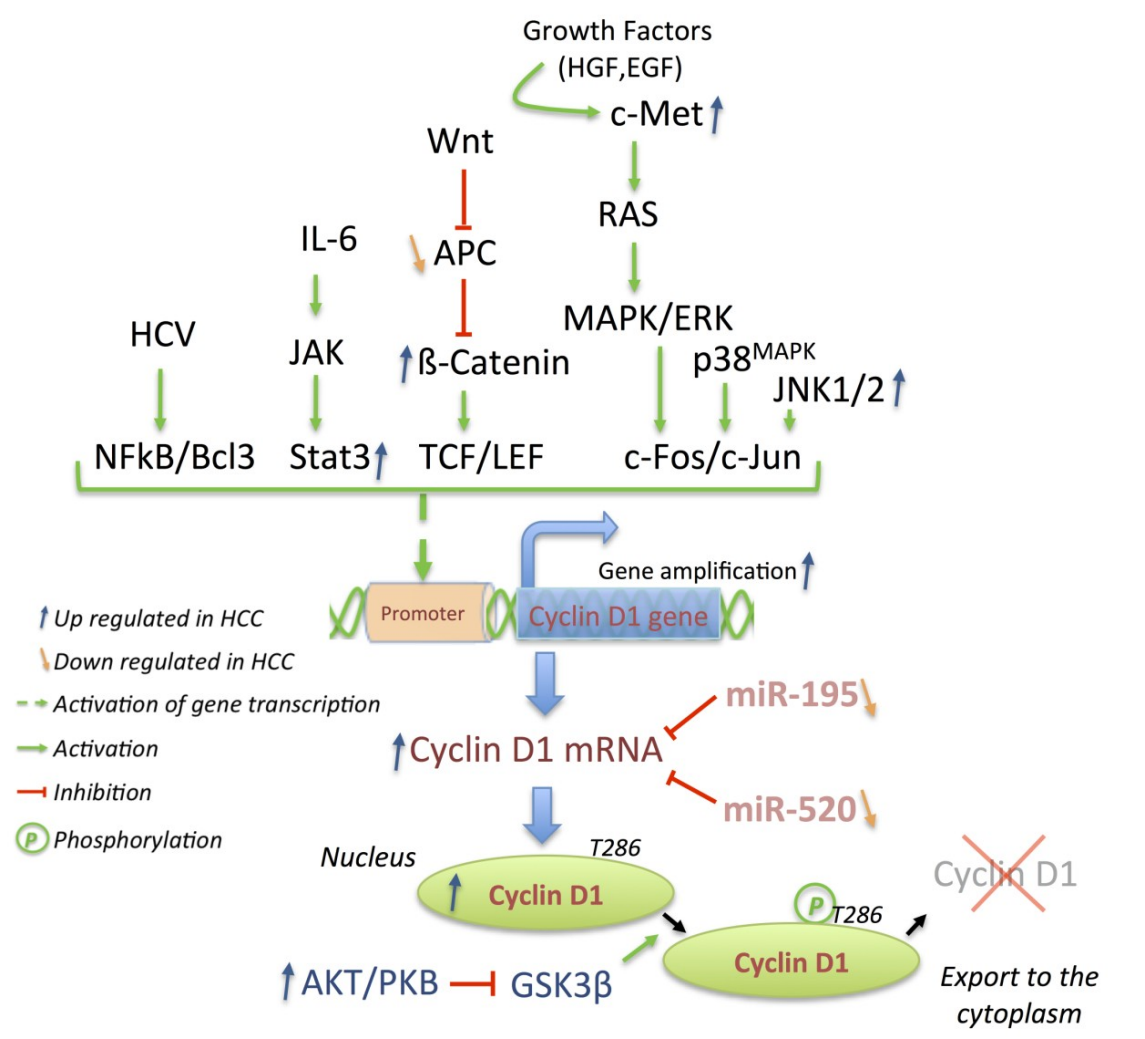
Cyclin D1 gene expression integrates mitogenic signals as well as their oncogenic alterations, which are frequently observed in HCC. MicroRNA miR-195 and -520 targeting cyclin D1 transcripts are down regulated in HCC. These alterations induce overexpression of the cyclin D1 protein in the nucleus due to absence of T286 phosphorylation, impairing its nuclear export as a result of increased level of Akt in HCC.

As in a number of human cancers, the four main cyclin types (D, E, A, B) have been observed to be overexpressed in HCC and are associated with different outcomes. Overexpression of cyclin D1 and cyclin E1 in HCC has been demonstrated by multiple approaches [122,123,124,125,126,127]. Although comparison of amplification frequencies from different studies remains arduous, human chromosome 11q13.2 containing the genes coding for cyclin D1 (CCDN1) is amplified in 11%–20% of HCC [124,125] whereas 19q12 amplifications (containing cyclin E1 [CCNE1]) are less frequent [126]. Cyclin D1 gene amplifications explain only partially the frequent cyclin D1 protein overexpression encountered in HCC. As a sensor of mitogenic signals as well as during oncogenesis, cyclin D1 transcription, translation, or stability is under the control of several pathways such as Wnt/β-catenin, growth factors/Ras/MAPK/Jun/Fos or GSK3β/AKT pathway, cytokines/Jak/Stat3 signaling or even NFκB (Figure 5) [128,129,130,131]. Despite the transcriptional activation of the cyclin D1 gene by nuclear β-catenin in normal or cancer cells [132,133], the link between β-catenin and cyclin D1 remains complex in HCC. Analyses of cyclin D1 transcript and protein level from human HCC samples have not shown a positive correlation between cyclin D1 expression and total or nuclear β-catenin as a result of amplification or mutation of its gene [134,135]. Conversely, overexpression of truncated β-catenin (DN90aa) or human c-Met (hMet) induces cyclin D1 expression in the liver of injected FVB/N mice but not in primary hepatocytes [136]. Similarly, mice expressing Ser45 mutant of β-catenin from the albumin promoter (S45A/D/F; phosphorylation site targeted by Ck1 priming β-catenin degradation), displayed an increased hepatic cyclin D1 expression after 1 month that was rapidly reduced in the absence of spontaneous tumor formation [137]. Surprisingly, mice overexpressing hMet and truncated β-catenin do not require cyclin D1 expression for tumor development, since it was shown that tumor can grow faster in its absence (cyclin D1^−/−^ mice). This can be potentially explained by compensatory expression and activity of cyclin D2 bound to Cdk6 and successive overexpression of cyclin E1 and B1 [136].

Overexpression of HGF, c-Met, Ras or BRAF transduces entirely or partially their signal through the MAPK cascade (Figure 5) [138,139,140,141]. As observed in HCC tissue samples or cell lines, this leads to an increased expression and phosphorylation of Erk1/2, p38 MAPK or even JNK1/2, resulting preferentially in elevated c-Fos activity and therefore expression of cyclin D1 and phosphorylation of pRb [52,142,143,144]. A similar correlation has been observed between the JAK/Stat3 pathway and cyclin D1 expression via constitutively activated Stat3 in a number of HCC tumors or cell lines [145,146,147].

Overexpression of cyclin D1 in HCC has also been observed in response to other pathways. Various actions and modulations of miRNAs have been recently reported in HCC [148,149] among which miR-195 and miR-520 are downregulated in HCC (Figure 5) [150,151]. When overexpressed in HCC cell lines, both of them have been reported to induce cell cycle arrest by targeting cyclin D1, Cdk6, or E2F3 [150,151]. Furthermore, the small HBx protein promotes cell proliferation via the activation of NFκB2/BCL-3 complexes, which mediates cyclin D1 overexpression [152]. The nuclear localization of cyclin D1 may be associated with increased proliferation. Indeed, constitutive activation of PI3K/AKT signaling in HCC favors nuclear localization of cyclin D1 via the inhibitory phosphorylation of GSK3β (Figure 5) [144,153,154]. However, according to different studies, overexpression of cyclin D1 has been correlated with poor differentiation and aggressiveness of HCC [123,127,155,156,157].

Transgenic overexpression of cyclin D1 in liver of C57BL/6 mice is sufficient to initiate hepatocellular carcinogenesis [158]. However, those mice displayed a slow transition from hepatomegaly, development of mitotic bodies and dysplastic cell nuclei progressing to liver dysplasia, adenoma and only after 17 months progression to HCC lesions [158]. The rare appearance of liver tumors in this model suggest that overexpression of cyclin D1 can be an initiating event but requires additional genetic alterations to trigger HCC development. Similarly to pRb inactivation, induced cyclin D1 overexpression in already established dysplasia would allow to accelerate tumor formation and proliferation.

In agreement with this model, overexpression of cyclin D1 is associated with chromosomal abnormalities [159,160,161]. *In vivo* expression of cyclin D1 after transient transfection using recombinant adenoviruses leads to apparent modifications of the mitotic spindle associated with supernumerary centrosomes. Many of the hepatocytes expressing cyclin D1 rapidly become polyploid (4N and 8N), mainly in the form of aneuploidy. Although the majority of these cells are eliminated by apoptosis after checkpoint activation, a portion resists and retains abnormal centrosome numbers [161].

Those latter observations could explain the slow appearance of HCC induced by persistent cyclin D1 overexpression but additional functions of cyclin D1 have been reported (see [131] for a detailed review). The phosphorylation of pocket proteins but also of various transcription factors as well as the non-catalytic activity of cyclin D1 by its ability to link a large panel of transcription factors, nuclear receptors, regulators of histone acetylation, and Rock pathway members with DNA damage response proteins, could affect HCC and explain the heterogeneity associated with cyclin D1 expression.

**Figure 6:**
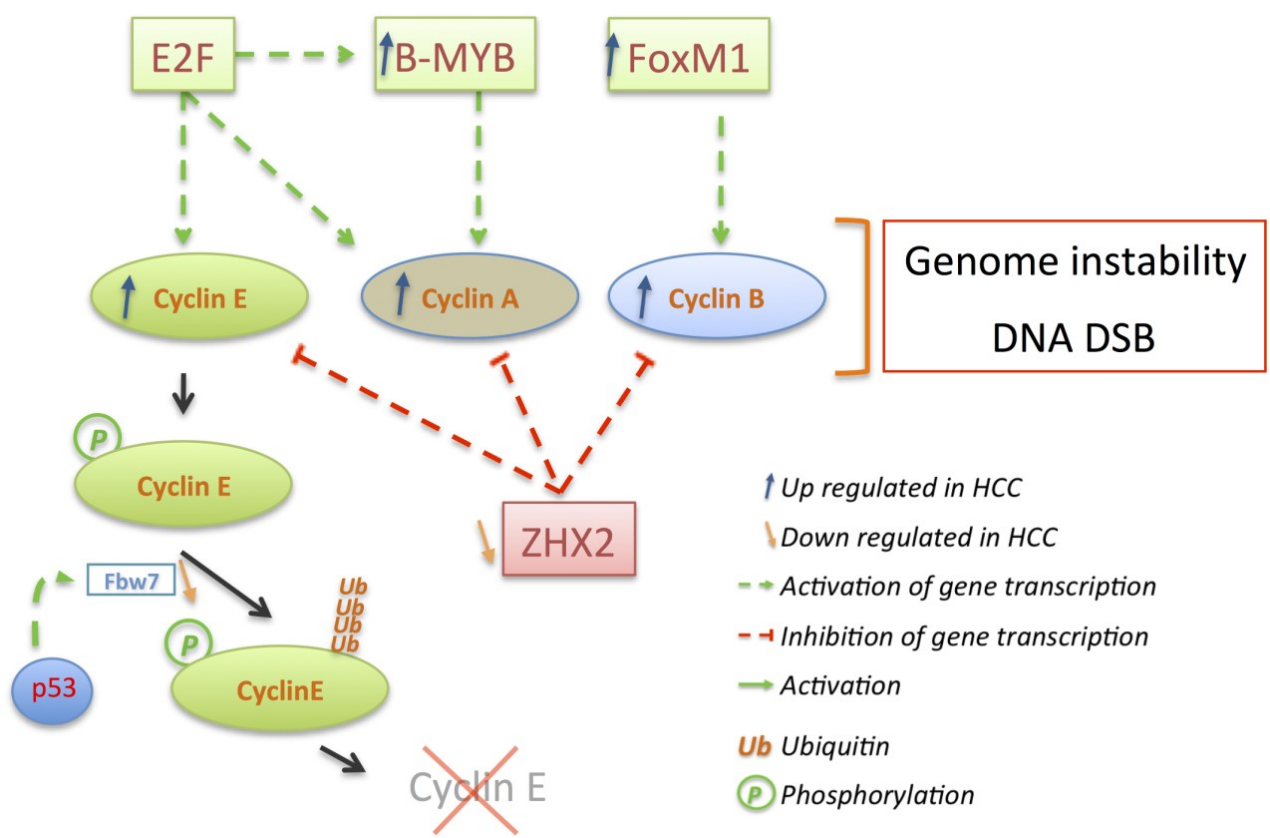
Cyclin E, A, and B overexpression triggers genome instability and DNA double strand breaks (DSB). E2F activity induces cyclin E, A, B expression as well as B-Myb expression. B-Myb in turn enhances expression of cyclin A while FoxM1 increases cyclin B1 expression. Expression of all three cyclins is repressed by ZHX2 (Zinc finger and homeoboxes 2) whose expression is substantially down regulated in HCC through hypermethylation of its promoter. Cyclin E degradation is also misregulated in HCC via down regulation of the ubiquitin ligase Fbw7, which is a p53 target gene.

The frequency of cyclin E overexpression is prevalent (≈65%–70% of HCC tested cases) and is induced by a variety of mechanisms independent of CCNE gene amplification shown in ≈10% of HCC samples tested by Jung *et al*. but not or less observed in other studies [126,162,163]. Under normal conditions, cyclin E is transcriptionally induced in late G1 by E2F1, which is activated by pRb de-repression (Figure 6). Similarly, ZHX2 (zinc fingers and homeoboxes 2) represses transcription of several genes including cyclin E and cyclin A, but is strongly deregulated in liver cancer due to hypermethylation of its promoter [164]. ZHX2 by its association to NF-YA binds to cyclin E and A promoters and represses their expression [165]. Although the effect of ZHX2 in HCC remains contradictory, a significant correlation has been suggested between reduced nuclear ZHX2 and poor overall survival due to increased hepatocytes proliferation [165]. Moreover, overexpression of ZHX2 in HCC cell lines reduces cyclin E and A expression and inhibits cell proliferation *in vitro* or *in vivo* in nude mice [165].

Parallel to its expression, cyclin E degradation can be impaired in HCC leading to increased protein expression (Figure 6). Ubiquitinated by SCF^Fbw7^ during S phase, cyclin E remains stably expressed in HCC and other cancers harboring markedly down regulation of the haploinsufficient tumor suppressor Fbw7 [166,167,168,169]. Fbw7 is subject to point mutations around its substrate-binding site (see [170] for a detailed Fbw7 review) but more importantly it is a p53 target gene, which is consequently deregulated in several HCC cell lines [169]. In those cells lines, through Fbw7 induction, transient expression of p53 prevents cyclin E accumulation leading to decreased proliferation and increased apoptosis [171]. On the other hand, loss of p53 correlates with cyclin E overexpression as has been previously observed in human HCC samples [156].

Depletion of cyclin E by siRNA in cyclin E overexpressing cells (Hep3B, HepG2, SNU449) indicated its importance by inducing growth arrest and apoptosis in contrast to cells with normal levels of cyclin E [172]. Similar results have been reported in primary cells obtained from DEN-induced mouse liver tumors where cyclin E silencing allows p53 *de novo* expression and activity inducing p21 and reducing anti-apoptotic Bcl-XL levels [173]. However, effects of cyclin E depletion seem to appear more efficient in cells with an altered p16^Ink4a^/pRb pathway [174,175]. Taken together, these results place cyclin E in HCC at the junction between the pRb and p53 pathways connected to proliferation and chromosome instability. Deregulation of cyclin E expression and aberrant activation of its Cdk complexes has been reported in different cancers to trigger genomic instability [176]. Moreover cyclin E and cyclin A, when overexpressed, have been reported to induce DNA double strand breaks in mammalian cells potentially via interfering with pre-replication complexes [177,178,179]. The proper timed regulation of cyclin E and A activities is critical because premature cyclin A expression results in impairment of DNA replication [180]. However, no rearrangement of the cyclin A locus has been observed despite the insertion of HBV in the cyclin A gene in one case [181]. Cyclin A accumulation has been reported in 39%–50% of tested HCC [182,183] and is associated with a increased percentage of cells in S and G2/M phases [184].

Like cyclin E, cyclin A and cyclin B1 integrate deregulations of the pRb pathway but are also under the control of other transcriptional regulators such as B-Myb or ZHX2 for cyclin A [165,185] or FoxM1 for cyclin B1 [186], which are found frequently deregulated in HCC (Figure 6).

### 3.3. p21 and p27: A Complex Connection

**Figure 7:**
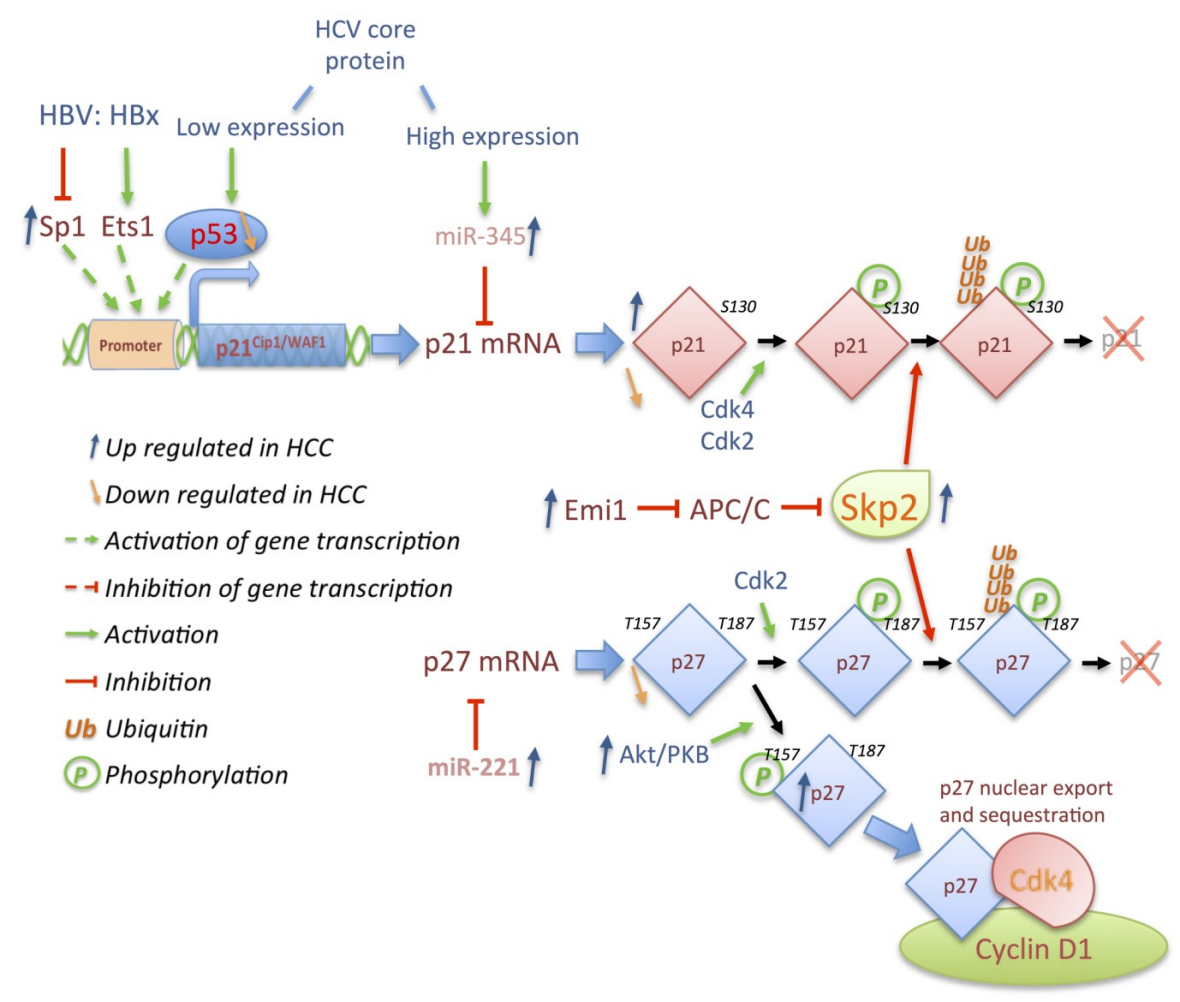
Cdk inhibitors p21 and p27 are deregulated in HCC. Low expression level of HCV expression core protein activates p53, which induces expression of p21 while high expression level of its mature form activates miR-345 targeting p21 mRNA. HBV X protein (HBx) results in bivalent actions on the transcription of the p21 gene by activating Ets1 or repressing Sp1 transcription factors. p27 mRNA is targeted by miR-221, which is often upregulated in HCC. p21 and p27 are phosphorylated by Cdk2 or Cdk4 respectively on S130 and T187 and ubiquitinated by Skp2 resulting in p21 and p27 degradation. Skp2 is targeted for degradation by the APC/C complex, itself regulated by Emi1, which is frequently overexpressed in HCC like Skp2. p27 is not only down regulated in HCC, its overexpression is a result of phosphorylation on T157 by Akt/PKB, leading to export of p27 to the cytoplasm where it is sequestered by Cdk4/cyclin D complexes.

Regulation, roles, and involvements of the Cdk inhibitors p21^Waf1/Cip1^ and p27^Kip1^ in HCC are complex and not fully understood (Figure 7). Several studies reported a decreased expression of both inhibitors in various human HCC samples, especially associated with advanced stage, weak differentiation, and poor prognosis of the pathology [157,187,188,189,190,191]. However, others reported that p21 and p27 can be found overexpressed, preferentially during early stages of hepatocarcinogenesis [188,192], contrasting with the idea that they act as indispensable negative regulators of G1/S and G2/M transitions. Gene transcription, mRNA, and protein expression of p21 and p27 are the targets of numerous regulatory mechanisms and oncogenic anomalies resulting in down regulation of p21 mRNA and overexpression of p21 protein, observed at the same time in HCC samples [188]. In addition to their altered expression, both inhibitors, although mostly localized and active in the nucleus, can be relocalized in the cytoplasm or sequestered in specific complexes potentially affecting other pathways (Rock/Cofilin, apoptosis, *etc*.).

p21 inhibits cell cycle progression in response to p53 activation and is often undetectable in HCC when p53 is mutated or deleted. However, p21 overexpression can be independent of p53 expression or induced through p53-independent pathways [187,189]. Among those pathways, hepatitis viruses maintain an ambiguous connection to the p21 expression. Depending on the expression system used, contradictory reports indicate that HCV core protein represses or enhances p21 expression (Figure 7) [193,194,195,196]. The HCV core protein is expressed firstly as a long cytoplasmic form of 191 amino acids (immature form), which later is cleaved to its mature, nuclear form of 173 amino acids [197,198,199]. Analyses using inducible expression systems demonstrated that this protein modulates p21 expression in a biphasic manner depending on the expression and the localization of its immature (p21 induction) or mature form (p21 repression) [197,199]. Whereas induction of p21 by HCV is largely dependent of p53 activity to prevent massive cell death, Shiu *et al*. recently highlighted repressive mechanisms of p21 expression through upregulation of miR-345 expression by the mature HCV core protein (Figure 7) [198]. Similar contradictory observations have been reported for HBV mediated by HBx expression levels [200]. Indeed, its expression in Hep3B cells transactivates p21 via Ets-1 binding to the p21 promoter [201,202] but can also repress p21 expression via decreases of Sp1 activity [203,204].

Taken together, these results reveal that p21 acts as a brake of the cell cycle and its down regulation is required for hepatocarcinogenesis induced by hepatitis viruses, mitogens (JNK, MAPK, …) [205] or toxic metabolites accumulation induced by tyrosinemia type 1 [206,207]. However, changes in p21 levels in response to liver injury depend among others on p53 activity, which determines liver regeneration or tumor development.

In contrast, the functions of p27 in HCC are well characterized. Loss of p27 provokes multiorgan hyperplasia, tumorigenesis and is associated with an increase of hepatocyte cell density in the liver [208,209,210]. Specific deletion of p27 in liver accelerates DNA replication after loss of extensive mass but does not induce spontaneous liver tumors [81]. Loss of p27 enhances tumor progression only in chronically injured livers [211], corroborating clinical observations that absent or reduced p27 expression is related to advanced stages and aggressiveness of HCC, and poor survival prognosis [157,190,212]. In this context, the mechanisms leading to lower expression of p27 in HCC are multifaceted. Like p16 or p21, p27 gene expression can be impaired by hypermethylation of its promoter but this is rare [213,214]. Its reduced expression occurs mostly at post-transcriptional and/or post-translational levels through overexpression of miRNA, ubiquitin ligase deregulation, or in connection with hepatitis viruses (Figure 7).

Among the different miRNA deregulated in HCC, miR-221 is up regulated in approximately 70% of HCC and positively correlates with advanced stage HCC, tumor size and metastasis [215,216,217]. miR-221 targets p27 mRNA as well as p57 mRNA, promoting HCC proliferation although the mechanisms remain unknown [215,217]. Despite miRNA actions, the major mechanism of p27 down regulation occurs through its degradation. Parallel to its essential cofactor Cks1, cytoplasmic and nuclear ubiquitin ligase Skp2 expression is increased during progression of HCC. Skp2 targets the three Cip/Kip family members (p21, p27, and p57) for degradation in tumors [214,218]. Skp2 itself is a target of ubiquitin-degradation cascade through recognition by the APC/C^Cdh1^, and is often deregulated in HCC (Figure 7) [219,220,221]. In addition, Skp2 expression is controlled by several transcription factors deregulated in HCC, downstream of various signaling pathways [222].

However, regulation of p27 is mostly dependent on phosphorylation. While phosphorylation on Thr187 by Cdk2/cyclin E complexes is essential for its ubiquitination and degradation, p27 is also phosphorylated by PKB/AKT on Thr157 in HCC, inducing its relocalization to the cytoplasm and impairing its negative effect on nuclear Cdk/cyclin complexes (Figure 7) [223,224]. Surprisingly, this latter mechanism indicates that p27 can remain highly expressed and is frequently associated with p16 loss in some HCC tumors. In this context, p27 does not impair Cdk2/cyclin E activity as it remains in the cytoplasm and is sequestered by Cdk4/cyclin D1 complexes [225].

## 4. Outlook and Conclusions

In HCC as well as in other cancers, cell cycle progression is deregulated by a large number of aberrations involving either cell cycle proteins or their regulators. The implicated alterations accelerate cell proliferation especially during advanced stage HCC but strangely direct deregulations of major cell cycle regulators towards unexpected effects. Inactivation of pRb and hyperactivity of Cdks creates genomic instability while p21 via its different binding partners also plays a role in the cell death response. This sets the stage for a number of cell cycle regulators and therefore cell cycle based therapies are considered to treat various cancers including HCC. In this context, inhibition of Cdk1 activity, like its loss in mice, prevents liver cancer development induced by activated Ras/loss of p53 [226]. Specific inhibition of Cdk1 prevents cell division without inhibiting liver regeneration or function. Despite the potential toxicity of Cdk1 inhibitors for cell types with high proliferation index (especially stem cells), this treatment would be conceivable if administrated specifically to the liver because healthy adult liver contains few proliferation cells and Cdk1 is not expressed. Other Cdks could be targets too, whereas PD-0332991 a Cdk4/6 inhibitor halts proliferation of hepatocytes and HCC xenografts *in vivo* [227] and is being tested in phase 2 clinical trials as replacement drug for patients intolerant to Sorafenib treatment. Other Cdk inhibitors could be used jointly in response to specific alterations. Cdk2 inhibitors such as roscovitine (Seliciclib) or its second generation (CR-8) could be used in cyclin E overexpressing tumors with an altered pRb and intact p53 pathways to induce apoptosis. The combination of Cdks inhibitors like Cdk4/6 and Cdk2 inhibitors as well as knockdown of Skp2 or other oncogenes can be envisaged to stop proliferation of HCC cells, since genetic ablation of these has been shown to elicit such effects.

However, due to the potential of hepatocytes to enter quiescence, to not divide and become polyploid, impairment of cell cycle progression and division via inhibitors directed against Cdks would stop HCC proliferation but unfortunately could create a dormant pool of cancerogenous cells. As has been tested in other cancers, a combination of Cdk inhibitors treatment with cytotoxic agents or radiotherapy could provide new possibilities since radiation affects cell cycle arrested cells quite different from proliferating cells. For example, Cdk4/6 or other Cdk inhibitors could be used in cells developing rapid drug resistance to synchronize tumors cells in the cell cycle and boost cell death by specific therapeutic agents [228,229].

In this regard, cell cycle based therapy provides new therapeutic approaches for HCC because these cells proliferate at high rate compared to healthy liver cells that are mostly quiescent. However, the heterogeneity of expression of cell cycle regulators makes the use of specific therapies more difficult and requires the development of molecular markers, as well as diagnostic markers associated with clinical and anatomo-pathological outcomes. It is therefore indispensable to define the dependency of tumor cells to Cdk activity in relation to the expression of pRb, p16, p21, or p27 Cdk inhibitors as well as to develop new or more specific Cdks inhibitors in order to provide alternative therapies for HCC or other cancers. Combination therapies could be paramount for treatment of HCC; with cell cycle therapies one of the fixed legs, one has to wonder which other pathway to be inhibited would be most effective. Time will tell.
